# Cure for Drunkenness

**Published:** 1880-04

**Authors:** 


### CURE FOR DRUNKENNESS.

No one who has once been a drunkard is ever safe from falling, this side the grave; it is a terrible truth, but it is a reality. He only is comparatively safe, who is in constant fear of falling.

We knew a Mrs. H., in our childhood, who finding her husband dead drunk one day, sewed him up in a sheet, and gave him a tremendous cowhiding. He never got drunk again; in this case it was the fear of the hide, and not of the fall. But one of the speediest rousers from a state of stupid, beastly intoxication we have ever read of is, to turn the brute on his right side, hold up his left arm, and pour a pitcher of cold water down his sleeve slowly. He will walk perfectly sober in five minutes. But this only cures until next time. We rather think that the raw hide is a more vivid remembrancer; and our old friend, cold water, must yield the palm this time.

We urge upon all parents, in view of the many incurable eye diseases, to caution their children against reading by twilight, that is, not before sunrise nor after sunset. It would be greatly better not to allow them to read or sew by any artificial light; but if that is unavoidable, let it be imperative that they cease by nine o'clock at night in summer, and by ten at farthest, in the winter. It is a most inexcusable folly, and will, sooner or later, bring its punishment, to read or sew by gas, or lamp, or candle light, and then sleep after daylight next morning, as a habit. To persons of all ages it is a most injurious practice.

